# Quantification of trivalent non-replicating rotavirus vaccine antigens in the presence of aluminum adjuvant

**DOI:** 10.1016/j.jim.2021.113056

**Published:** 2021-07

**Authors:** David McAdams, Kyle Lakatos, Marcus Estrada, Dexiang Chen, Brian Plikaytis, Robert Sitrin, Jessica A. White

**Affiliations:** a Medical Devices and Health Technologies Global Program, Formulation Technologies, PATH, Seattle, WA, USA; b Biostat Consulting LLC, Big Canoe, GA, USA; c The Center for Vaccine Innovation and Access, PATH, Washington, DC, USA

**Keywords:** Rotavirus, Nonreplicating rotavirus vaccine, NRRV, CI, confidence interval, CV, coefficient of variation, DPBS, Dulbecco's phosphate buffered saline, ELISA, enzyme-linked immunosorbent assay, HRP, horseradish peroxidase, mAb, monoclonal antibody, NRRV, nonreplicating rotavirus, TBS, tris(hydroxymethyl)aminomethane buffered saline, T-20, Tween 20

## Abstract

Parenterally administered rotavirus vaccines may overcome the low efficacy observed in resource-poor regions that use live oral formulations. We have reported work on a trivalent nonreplicating rotavirus vaccine (NRRV) for parenteral administration consisting of the recombinant tetanus toxoid P2 CD4 epitope fused to a truncated VP8* fragment (P2-VP8*) for the P[4], P[6], and P[8] serotypes of rotavirus adjuvanted with aluminum. An essential part of developing this vaccine candidate was devising quantification methods for each antigen in the trivalent NRRV in the presence of aluminum adjuvant. This report describes the development of quantitative inhibition enzyme-linked immunosorbent assays (ELISAs) for in vitro antigenicity determination of the adjuvanted trivalent NRRV using serotype-specific monoclonal antibodies (mAbs) against each of the P2-VP8* antigens. Adjuvanted trivalent vaccine samples are titrated and incubated with a constant concentration of specific mAbs against each NRRV P2-VP8* antigen variant. Unbound mAbs are measured by ELISA to indirectly quantify the amount of each antigen present in the trivalent vaccine. Sensitive, specific, and reproducible inhibition ELISAs were developed and qualified for each antigen and used for final product quantification and release testing without desorption of the vaccine antigen.

## 1 Introduction

Rotavirus is a major cause of morbidity and mortality (GBD Diarrhoeal Diseases Collaborators, 2017; Tate et al., 2016) especially in young children. Several live oral rotavirus vaccines are in use, but their effectiveness is reduced in low- to middle-income countries compared with high-income regions (Parker et al., 2018a; Parker et al., 2018b; Velasquez et al., 2018). This phenomenon has been noted for other live oral vaccines, such as polio and cholera, but the reasons for it are uncertain (Church et al., 2018). Recently, attention has been focused on development of parenterally administered vaccines in an effort to circumvent the decreased efficacy of oral vaccines. Nonreplicating rotavirus vaccine (NRRV) is one of the approaches for a parenteral vaccine (Kirkwood et al., 2019; Wen et al., 2012).

During rotavirus infection, one of the structural proteins of the virion is cleaved via proteolysis into two peptides, VP5 and VP8, which are required for cellular infection (Midthun and Kapikian, 1996). The rotavirus strains that most frequently infect humans can be grouped into three P serotypes, P[4], P[6], and P[8], each defined by slightly different VP8 peptide structures (Wen et al., 2012). Our recently reported trivalent NRRV vaccine formulation (Lakatos et al., 2020) consists of these three variants, each fused with the P2 epitope of tetanus toxoid and adsorbed onto an aluminum hydroxide adjuvant (Wen et al., 2014). This vaccine candidate is currently being evaluated in human clinical trials (Groome et al., 2017; Fix et al., 2015).

The goal of the present study was to develop sensitive and serotype-specific methods for quantification of each of the three P2-VP8* antigens in the NRRV candidate adjuvanted with aluminum hydroxide. While many adjuvanted vaccine assays include a step to desorb the antigen from the adjuvant before quantification (Rossi et al., 2020), desorption of the P2-VP8* antigen from the adjuvant particles was found to be inefficient or could potentially introduce structural changes in the antigen (Lakatos et al., 2020). To address this, we sought to develop an assay to evaluate complete, native vaccine formulation, consisting of antigen adsorbed to the aluminum adjuvant without an antigen desorption step. The final target criteria for the assay development was to develop assays that were specific for each of the individual antigens present in the trivalent vaccine in the presence of aluminum adjuvant with a quantitative range below 50% of the target antigen dose of 30 μg per antigen. As limited vaccine lots had been manufactured at the time of assay development the specification for potency was set at 50%–150% until more data become available. This specification was used for assay development to ensure sensitivity to the antigen range being evaluated in human clinical studies. This report describes the development of quantitative ELISAs to measure antigen content and antigenicity in an adjuvanted trivalent NRRV candidate, which is essential for both in-process and final vaccine product analysis and release (Agarwal et al., 2020).

## 2 Materials and methods

Assay buffers were screened and selected based on previous ELISA development by our laboratory (White et al., 2017). Initial experiments used a block and assay buffer consisting of 5% nonfat dried milk (Quality Biological), 0.5 mM Dulbecco's phosphate buffered saline (DPBS, Cytiva, formerly GE Healthcare Life Sciences), and 0.05% Tween® 20 (T-20, Fisher). Later experiments were conducted with a commercially available blocking buffer of 1% Hammarsten grade casein in tris(hydroxymethyl)aminomethane buffered saline (TBS, Thermo Scientific) with the addition of 0.05% T-20. Bridging studies were conducted to verify that changing the assay buffer had not impacted the previously identified assay conditions, as described below.

### 2.1 Monovalent standard and trivalent reference samples for assay development

Trivalent and monovalent samples were prepared for assay development using bulk NRRV monovalent antigens provided by two manufacturers. Walter Reed Army Institute of Research (Silver Spring, MD) monovalent antigens P2-VP8 P[4], P[6], and P[8] were provided at the following concentrations: P[4]: 0.90 mg/mL; P[6]: 0.83 mg/mL; P[8]: 0.94 mg/mL. SK Bioscience (South Korea) provided antigens at the following concentrations: P[4]: 0.69 mg/mL; P[6]: 0.59 mg/mL; P[8]: 1.09 mg/mL. Bulk antigen concentrations were determined by measuring absorbance at 280 nm (A_280_) for the purified antigens using variant specific calculated extinction coefficients. Bulk antigen concentrations were confirmed prior to use in assay development by A_280_. To limit potential loss during storage bulk antigens were thawed and prepared for each experiment. Assay development was conducted using both monovalent, bivalent, and trivalent vaccine material with and without the presence of the aluminum adjuvant. Bulk monovalent materials were mixed at a concentrations of 30 μg per antigen per 0.5 mL dose to achieve mixtures of unabsorbed monovalent, bivalent, or trivalent materials (60 μg per antigen per mL) and at 60 μg per antigen per 0.5 mL dose to achieve mixtures of unabsorbed monovalent, bivalent, or trivalent materials (120 μg per antigen per mL). Additional adjuvanted monovalent, bivalent, or trivalent antigen mixtures were prepared by adsorption to Alhydrogel® (Al(OH)_3_, Brenntag Nordic A/S, Accurate Chemical & Scientific Corporation) at 1.12 mg Al/mL overnight at 2 °C–8 °C with gentle mixing.

### 2.2 Monoclonal antibody generation and production

Precision Antibody™ (Columbia, MD) produced monoclonal antibodies (mAbs) against each of the NRRV antigens in the trivalent vaccine (P[4], P[6], and P[8]). A total of three 6–7-week-old BALB/c mice were immunized with a combination of P2-fused and non-fused antigens and received multiple booster immunizations until an acceptable antibody titer was achieved. Primary hybridoma screening was performed against each homologous antigen target VP8*, P2-VP8*, and P2 alone. Confirmatory screening (by direct ELISA and dot blot) was performed against heterologous antigens. Hybridomas were selected based on specificity for the homologous antigen (with and without the P2 fusion), lack of reactivity to heterologous antigens, and production efficiency measured as supernatant protein concentration.

### 2.3 Preliminary assay development

#### 2.3.1 Monoclonal antibody dilution screening

To measure the sensitivity of each of the VP8* specific monoclonal antibodies, a dilution curve was tested using a direct ELISA format. Each antigen was coated onto ELISA plates at 1 μg/mL in DPBS. Dilutions of the relevant mAbs started at 4 μg/mL and were halved in each successive dilution. Bound mAb on high-binding ELISA plates (Corning) was detected with horseradish peroxidase (HRP)-labeled goat anti-mouse immunoglobulin G secondary antibody (Millipore). The secondary antibody was added at a dilution of either 1:2000 or 1:4000. Reaction of the HRP with 100 μL/well tetramethylbenzidine (Sigma) substrate was stopped with 100 μL/well of a 1 M sulfuric acid fixative solution (Sigma). Absorbance was measured at 450 nm using an ELISA plate reader (Molecular Devices).

#### 2.3.2 Monoclonal antibody selectivity

To demonstrate selectivity of the mAbs and the ability to bind to antigen in solution, preliminary inhibition ELISAs were conducted for each of the three antigens. Each of the ELISAs included samples of homologous non-adsorbed and adsorbed monovalent antigen, non-adsorbed heterologous antigens, and trivalent adsorbed vaccine.

ELISA plates were coated with 100 μL/well of a 1 μg/mL solution of monovalent bulk P2-VP8* antigen diluted in DPBS. Coated plates were held at 2 °C–8 °C overnight. The following day, all standards and samples were blocked with 5% nonfat milk and placed on a rocker for 1 h at room temperature, to prevent detection antibody binding to the Alhydrogel. In a separate deep well plate (VWR), serial dilutions of monovalent standards and test samples were prepared in 5% nonfat milk in DPBS. Each mAb was prepared at twice the needed final concentration (identified in the prior dilution screening experiments) in 5% nonfat milk in DPBS with 0.1% T-20. The mAb dilutions were mixed in equal volumes with the diluted monovalent antigen standards and test samples in the deep well plates. The plates were sealed and covered with foil to protect from light and incubated overnight at room temperature (21 °C–25 °C) on a plate shaker (Thermo Scientific) at 500 rpm.

After incubation, the antigen-coated ELISA plates were retrieved from the 2 °C–8 °C refrigerator, washed three times with 300 μL of DPBS with 0.05% T-20 using a plate washer (Bioteck 405 TS) and blocked with 200 μL/well of 5% nonfat milk in DPBS with 0.1% T-20 for 1 h at room temperature. After blocking, the wash step was repeated.

The deep well plates were centrifuged at 2000 RCF for 10 min (Sorvall T6000B). mAb bound to antigen adsorbed to Alhydrogel remained in the pellet after centrifugation and was not detected in the ELISA. The remaining unbound mAb in the supernatant was transferred and quantified on the ELISA plate using HRP-labeled secondary goat anti-mouse immunoglobulin G. Antibodies that reacted specifically with the homologous antigen in solution were selected for further inhibition assay optimization.

#### 2.3.3 Assay quantification and interference studies

Quantification was first evaluated using interference studies, where individual components of the proposed vaccine were selectively compared to measure any nonspecific loss or gain of signal. This was done by testing a combination of monovalent, bivalent, and trivalent antigen mixtures with and without adjuvant.

Materials were prepared from purified, monovalent bulk antigens to make a soluble trivalent antigen mixture of 240 μg/mL/antigen (720 μg/mL total antigen). The trivalent mixture was then mixed equally with a 2× Alhydrogel solution (2.24 mg Al/mL) to achieve a test sample with a final concentration of 120 μg/mL/antigen (P2-VP8 P[4], P[6], P[8] for 360 μg/mL total antigen) on Alhydrogel at 1.12 mg Al/mL. Mixtures of monovalent and bivalent antigen test samples were also prepared using the above dilution scheme. Monovalent unadjuvanted and adjuvanted P2-VP8 P[4], P[6], and P[8] antigens prepared from the same stocks were used as comparators for the quantification of interference. This initial antigen concentration was selected based on the highest dose evaluated in human clinical studies (Groome et al., 2017). Once the clinical dose of 30 μg/dose/antigen or 60 μg/mL/antigen (P2-VP8 P[4], P[6], P[8] for 180 μg/mL total antigen) was selected final trivalent materials were prepared for evaluation in the developed ELISA method using this clinical concentration. Materials were prepared from purified, monovalent bulk antigens to make a soluble trivalent antigen mixture of 120 μg/mL/antigen (360 μg/mL total antigen). The trivalent mixture was then mixed equally with a 2× Alhydrogel solution (2.24 mg Al/mL) to achieve a test sample with a final concentration of 60 μg/mL/antigen (P2-VP8 P[4], P[6], P[8] for 180 μg/mL total antigen) on Alhydrogel at 1.12 mg Al/mL.

### 2.4 Final assay qualification

#### 2.4.1 Blocking agent optimization

As noted above, all initial experiments were conducted using nonfat dry milk in the assay buffer (DPBS). However, milk powder is not standardized (Galva et al., 2012) from lot to lot, resulting in variability and potential interference with the antigens when different sources are used. To reduce this added variability, alternate blocking buffers were screened, including bovine serum albumin, sodium caseinate, Hammarsten grade casein, fish gelatin, and type B gelatin. The final buffer selected was a commercially available 1% Hammarsten grade casein in TBS, with the addition of 0.05% T-20. This buffer provided low background, acceptable sensitivity, and availability of a uniform product. Bridging studies were then conducted to ensure antigen specificity and assay sensitivity were not impacted by using this buffer.

#### 2.4.2 Antigen content

The concentration of each antigen in the test sample was determined by comparing unknown test samples to a trivalent P2-VP8* standard. A 4-parameter logistic regression analysis was performed to generate a standard curve from the 450 nm absorbance values of the trivalent standard using SoftMax® Pro software. For a standard curve to be used for interpolation the R^2^ value must be greater than or equal to 0.990. The antigen values for the unknown test samples were then interpolated by applying the standard curve to the absorbance values for the unknown test samples. Unknown test samples were tested in triplicate and values averaged to generate final antigen concentrations.

#### 2.4.3 Statistical methods

Inhibition assays for each of the P2-VP8* antigens were performed by two operators on three different days. On each day, each operator ran three plates for each antigen. Assay parameters as outlined in Table 1 included limit of detection, limit of quantification, linear range and the R^2^ of the standard curve. The limit of detect and quantification were calculated based on ICH guidelines using the standard deviation of the blank. These assay parameters may be further refined once the method has been transferred to a manufacturing partner and validation has been completed. Concentrations were used to measure within- and between- sample, plate, and operator variances. A mixed-model analysis of variance was used to quantify these variances, expressed as coefficients of variation (CV). Sample, plate, and operator were modeled as random effects. Accuracy was calculated by comparing means from the mixed models to the starting variant antigen concentration for standard and test samples (60 μg/mL).

**Table 1:** Final inhibition assay format for NRRV P2-VP8* antigens.

Inhibition ELISA parameters	Final assay formats
Starting variant antigen concentration for standard and test samples	60 μg/mL per antigen
Dilution across plate	2:3 (1.5-fold)
Primary monoclonal antibody	P[4]: 0.50 μg/mL
	P[6]: 0.12 μg/mL
	P[8]: 0.06 μg/mL
Assay buffer	1% Hammarsten grade casein in TBS + 0.05% Tween-20
Secondary antibody	1:4000
Tetramethylbenzidine incubation time	15 min
Linear range	Approximately 0.4–17 μg/mL
Limit of detection	0.05–60 μg/mL
Limit of Quantification	1–17 μg/mL
R^2^ of standard curve	≥ 0.990

## 3 Results

### 3.1 Monoclonal antibody screening and characterization

From the *initial screening* of mAbs, clones were selected that were specific to each VP8* antigen and P2-VP8* antigen and lacked reactivity to the P2 epitope alone as determined by a direct ELISA. Of the mAbs produced for potential use, three were selected for P[4], three for P[6], and four for P[8]. One lead mAb was then chosen for each antigen based on sensitivity and production efficiency from the hybridoma culture.

In *dilution screening*, the last dilution in the plateau portion of the curve was selected as the working concentration of the mAb for further testing. All the antibodies exhibited full saturation in both secondary antibody dilutions screened, so the 1:4000 dilution was chosen for further use.

In *selectivity studies*, none of the variant-specific mAbs selected showed cross-reactivity with any of the heterologous antigens or the adjuvant in the inhibition ELISA format (data not shown). Dilution curves were screened to identify an appropriate starting concentration, dilution amount, and number of dilutions needed to ensure a complete curve was generated for interpolation. For P[4], a two-fold working solution of 1.50 μg/mL was appropriate. For P[6], we used 0.12 μg/mL, and for P[8], 0.60 μg/mL, with protein concentrations determined by A_280_ measurements.

**Fig. 1:**
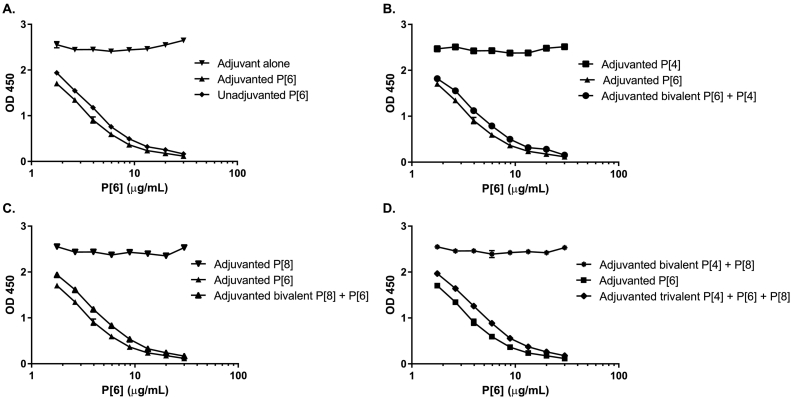
P[6] specific mAb specificity study results. (A) Presence of unadjuvanted monovalent P[6] antigen, adjuvanted monovalent P[6], and aluminum adjuvant alone. (B) Presence of adjuvanted monovalent P[4], adjuvanted monovalent P[6] antigen, and adjuvanted bivalent P[4] and P[6] antigens. (C) Presence of adjuvanted monovalent P[6], adjuvanted monovalent P[8] antigen, and adjuvanted bivalent P[6] and P[8] antigens. (D) Presence of adjuvanted monovalent P[6], adjuvanted bivalent P[4] and P[8] antigens, and adjuvanted trivalent vaccine. Representative data from two samples tested in triplicate experiments.

In *interference studies*, the presence of heterologous antigens or the adjuvant did not interfere with the quantification of the individual P[4], P[6], or P[8] assays (representative data shown in Fig. 1). A slight offset was observed between adjuvanted and unadjuvanted test samples. This could be due to the availability of additional epitopes which are blocked from mAb binding when the antigen is bound to adjuvant. While the change in concentration caused by this offset was minor it was decided to move forward with an adjuvanted standard for vaccine quantification.

#### 3.1.1 Bridging studies

Significant variability was observed after transfer of research methods to an alternate testing site. Variations in the source of the milk were identified as the cause of this variability, so later experiments were conducted with a commercially available blocking buffer. A block consisting of 1% (*w*/*v*) Hammarsten grade casein in TBS buffer was selected as the blocking reagent for use in the final assay format. The antigen specificity of each assay was confirmed using the casein blocking buffer compared to the previous milk buffer (data not shown). After selection, testing with and without the addition of 0.05% (w/v) T-20 to the casein blocker was evaluated for each antigen-specific assay. The addition of T-20 appeared to reduce the sample-to-sample variability and was selected for the final assay format (data not shown).

**Fig. 2:**
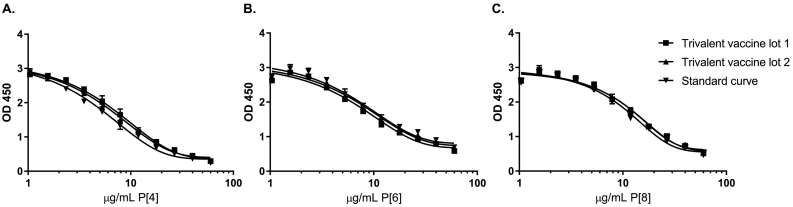
Representative ELISA results from two operators. Testing of two trivalent adjuvanted vaccine lots and standards is shown for all three antigen assays: (A) P2-VP8 P[4] antigen content, (B) P2-VP8 P[6] antigen content, and (C) P2-VP8 P[8] antigen content. Results show replicate results from two operators testing two separate vaccine lot test samples. R^2^ > 0.99 for all three graphs shown.

Based on all testing, the final assay conditions were selected as shown in Table 1. Assay parameters represent qualification of the methods and will be confirmed after transfer to a manufacturing partner for validation. Representative ELISA results are shown in Fig. 2 (replicate results from two operators).

### 3.2 Analysis of variance results

To assess the different levels of variability, the CV was calculated using concentration data for the samples in the specimen panel (Table 2). Within-operator, plate, and day CVs were all <10%. Between-operator, plate, and day as well as repeatability CVs were also all <10%. While the assays from each operator underestimated the given starting antigen concentration for test samples as slightly less than 60 μg/mL, all the values were within the variability of the test method. The protein concentration of 60 μg/mL was determined from the A_280_ measurement of the bulk vaccine antigen after theoretical dilution and was not remeasured at this low concentration due to the limited accuracy of this protein measurement method at lower concentrations. Additional testing is being conducted to better understand the correlations between the ELISA result and the measure of protein concentration by A_280_ and bicinchoninic acid methods.

**Table 2:** NRRV assay reproducibility for two operators.

		Antigen	P[4]		P[6]		P[8]	
		Operator	1	2	1	2	1	2
Statistical analysis	Concentration (μg/mL) Average ± SE (% expected)		51.4 ± 1.3 (86%)	53.8 ± 1.3 (90%)	53.1 ± 0.9 (89%)	58.8 ± 1.9 (98%)	57.5 ± 2.0 (96%)	58.4 ± 0.9 (97%)
	Overall concentration (μg/mL) average ± 95% CI	Mean	52.21		55.96		57.94	
		Lower 95% CI	44.63		35.45		50.78	
		Upper 95% CI	59.79		76.47		65.10	
	Within effects	Plate CV	7.62%		9.01%		7.75%	
		Day CV	8.00%		9.01%		6.33%	
		Operator CV	8.88%		8.02%		7.80%	
	Between effects	Plate CV	8.89%		7.86%		6.68%	
		Day CV	8.13%		7.36%		8.19%	
		Operator CV	7.50%		8.98%		6.27%	
		Repeatability	7.15%		6.24%		6.27%	
		Accuracy	87.02%		93.27%		96.57%	

CI, confidence interval; CV, coefficient of variation; SE, standard error. Duplicate samples were tested in triplicate by two operators.

### 3.3 Antigen quantification

Antigen quantification using an unadjuvanted monovalent standard, an unadjuvanted trivalent reference standard, and an adjuvanted trivalent standard were compared, and difference was observed in the final concentration calculated (data not shown). An adjuvanted trivalent standard was selected as the final assay standard for interpolation due to its similarity to the final vaccine analyte.

## 4 Conclusions

Three inhibition ELISAs that allow for quantification of the three rotavirus antigens present in the final adjuvanted trivalent NRRV were developed in this study. These assays do not require desorption of the antigen from the aluminum hydroxide adjuvant, which could introduce variability, depending on the desorption efficiency and potential for antigen degradation during the process. Successful production of this vaccine product by the manufacturer requires assays for testing both in-process samples and final product release. Rigorous selection criteria for serotype-specific mAbs combined with stringent assay criteria performance resulted in highly reproducible assays.

The assays developed in this study are quantitative assays that allow for determination of antigen content in an adjuvanted trivalent vaccine and are being used to release vaccine material. However, the assays developed are considered measures of antigenicity and not traditional potency assays, as the antigen-specific mAbs do not recognize a neutralizing epitope which allows for correlation to a relevant immunologic endpoint. We were unable to identify neutralizing antibodies that were also antigen variant-specific, which is likely due to the high degree of homology between each of the antigens (~65%–85%) present in the trivalent vaccine. In addition to the monoclonal antibodies developed under this project, we investigated neutralizing antibodies kindly provided by Stanford University and Royal Children's Hospital Australia for screening for antigen specificity (Nair et al., 2017; Coulson, 1993), but none were identified that were both antigen specific and neutralizing for the VP8* peptide fragments represented in this trivalent vaccine formulation. To further support use of these methods to evaluate vaccine antigenicity and release, additional work is in progress to evaluate the correlation between the inhibition ELISAs developed and animal model immunogenicity. These findings are being drafted in a manuscript by the project team and will be submitted for publication very soon (McAdams, D et al.).

In conclusion, we have demonstrated an inhibition ELISA methodology that is sensitive to relevant clinical concentrations, specific for each NRRV P2-VP8* antigen, and reproducible for each NRRV P2-VP8* antigen variant, for final drug product quantification and release testing in the presence of an aluminum adjuvant.

## Disclosure statement

No potential conflict of interest is reported by the authors.

## Authors' contributions

DM, KL, ME, and JW completed work and drafted the manuscript. DC and RS reviewed data and provided technical guidance in addition to reviewing the manuscript. BP completed statistical analysis.
